# Clinically sound and person centred: streamlining clinical decision support guidance for multiple long-term condition care

**DOI:** 10.1136/bmjgh-2023-013816

**Published:** 2024-10-28

**Authors:** Ruth Vania Cornick, Inge Petersen, Naomi S Levitt, Tamara Kredo, Vanessa Mudaly, Carol Cragg, Neal David, Tasneem Kathree, Mareike Rabe, Ajibola Awotiwon, Robyn Leigh Curran, Lara R Fairall

**Affiliations:** 1 Knowledge Translation Unit, Department of Medicine, University of Cape Town, Cape Town, South Africa; 2 Centre for Rural Health, University of KwaZuluNatal, Durban, South Africa; 3 University of Cape Town, Cape Town, Western Cape, South Africa; 4 South African Medical Research Council, Cape Town, South Africa; 5 Department of Health and Wellness, Western Cape Provincial Government, Cape Town, Western Cape, South Africa; 6 School of Life Course & Population Sciences, King's College London, London, UK

**Keywords:** Other diagnostic or tool, Decision Making, Global Health, Health systems

## Abstract

The care of people with multiple long-term conditions (MLTCs) is complex and time-consuming, often denying them the agency to self-manage their conditions—or for the clinician they visit to provide streamlined, person-centred care. We reconfigured The Practical Approach to Care Kit, our established, evidence-based, policy-aligned clinical decision support tool for low-resource primary care settings, to provide consolidated clinical guidance for a patient journey through a primary care facility. This places the patient at the centre of that journey and shifts the screening, monitoring and health education activities of multimorbidity care more equitably among the members of the primary care team. This work forms part of a study called ENHANCE, exploring how best to streamline MLTC care in South Africa with its high burden of communicable, non-communicable and mental health conditions. This practice paper describes the four steps of codeveloping this clinical decision support tool for eleven common long-term conditions with local stakeholders (deciding the approach, constructing the content, clinical editing, and design and formatting) along with the features of the tool designed to facilitate its usability at point of care. The process highlighted tensions around prioritising one condition over another, curative over preventive treatment and pharmacological therapies over advice-giving, along with the challenges of balancing the large volume of content with a person-centred approach. If successful, the tool could augment the response to MLTC care in South Africa and other low-resource settings. In addition, our development process may contribute to scant literature around methodologies for clinical decision support development.

**SUMMARY BOX**

- Multiple long-term condition (MLTC) care is overwhelming for patients and providers.
- Clinical decision support is generally arranged per condition. There is a dearth of consolidated, person-centred clinical decision support for MLTC care.
- The Practical Approach to Care Kit (PACK) is a comprehensive evidence-informed, policy-aligned clinical decision support tool used in primary care in South Africa and several other low-income and middle-income countries (LMICs).
- We consolidated PACK recommendations for 11 long-term conditions to create a tool called the ENHANCE guide that would support a simplified, streamlined and person-centred MLTC primary care consultation.
- The ENHANCE tool forms part of a multifaceted intervention and study evaluating how best to streamline MLTC care in South Africa.
- If effective, the tool could augment current clinical decision support offerings to support South Africa’s and other LMIC responses to the MLTC burden.

## Making multiple long-term condition care more streamlined and person centred by reconfiguring clinical decision support

 South Africa is a middle-income country with a growing burden of multimorbidity, defined as the presence of two or more long-term conditions.^1^ Local multimorbidity prevalence rates are 30%–87% in older adults.^2^ Hypertension and HIV drive multimorbidity—common clusters are hypertension and arthritis, hypertension and diabetes, and hypertension and HIV. Chronic obstructive pulmonary disease (COPD), stroke and ischaemic heart disease are usually multimorbid.^3^

For the person with multiple long-term conditions (MLTCs)—the term preferred by people with multimorbidity^4^—the burden of medication, time, cost and health-related activities is considerable.^5 6^ The clinician’s workload is similarly overwhelming.^7^ However, clinical practice guidelines typically focus on a single disease and while they may mention comorbidities, detail about symptom overlap and medication interactions is limited.^8^ For example, approximately 86% of people with COPD globally have MLTCs^9 10^ yet the 2023 GOLD guideline recommends attention ‘to ensure simplicity of treatment and minimise polypharmacy’^11^ but lacks guidance on how to do so.

The WHO Technical Series on Safer Primary Care acknowledges that all clinical guidelines should consider multimorbidity.^12^ A few guidelines focus specifically on it. The UK National Institute for Health and Care Excellence published a guideline that provides principles for multimorbidity care. However, it lacks clinical detail for common multimorbidity configurations, focuses on older patients (in low-income and middle-income countries (LMICs) people with MLTCs are younger) and has a 56-item baseline assessment too lengthy for point-of-care use.^13^ The BMJ Best Practice Comorbidities Manager provides appropriate clinical detail but is designed for acute hospital settings in high-income countries.^14^

The Practical Approach to Care Kit (PACK) was developed by our knowledge translation unit (KTU) in South Africa. It is a clinical decision support tool that supports evidence-based, policy-aligned primary healthcare (PHC) in LMICs.^15 16^ Studies show a positive impact on a range of health outcomes and quality of care indicators across communicable and non-communicable conditions, and it is well received by end-users.^17^,^23^ Unlike most clinical guidance, PACK has a comprehensive scope, containing over 500 symptoms and conditions, including 24 conditions requiring long-term care. Across these conditions, there are 250 assessment (history, examination and investigations) recommendations, 136 advice-giving recommendations and 98 treatment (non-pharmacological and pharmacological) recommendations. However, PACK falls short of MLTC care because it still guides providers to address one condition at a time. This is onerous on providers and patients because time is limited in busy PHC settings, and inefficient if items are repeated (blood pressure checks for hypertension and diabetes) or incompatible (medication interactions or diagnostic thresholds differ with comorbidity). PACK’s checklists can also limit a person-centred approach. This is a key component of MLTC care, with the patient regarded as an equal partner.^24^

### Setting

Over 80% of South Africans attend public sector services. These services are free and delivered via 3500 PHC clinics and community health centres, supported by district hospitals for upward referrals and community health worker teams who visit households. Nurses form the foundation of clinical services supported by PHC doctors and limited specialist services. Rising multimorbid conditions led the South African Department of Health to develop the Integrated Clinical Services Management strategy to shift from disease-specific services towards integrated PHC services.^25 26^ PACK is a core component of this strategy. Multimorbidity is typical in older adults^2^ and long-term conditions place a high burden on PHC services. Clinical consultations are on average 7 minutes^27^ and are usually limited to renewing hand-written prescriptions and, where pharmacy resources are scarce, dispensing free chronic medications. Efforts to risk-stratify patients (for more intensive follow-up based on long-term condition control) or ensure continuity of care (patients seeing the same clinician at each visit) are non-standardised local initiatives.

### Supporting patients and providers in MLTC care

The Evidence-led co-created HeAlth systems interventioNs for MLTC-M CarE (ENHANCE) study is a multicentre 5-year National Institute for Health and Care Research-funded project in two South African provinces. It aims to codevelop and evaluate a multifaceted intervention for PHC to streamline MLTC care.

The ENHANCE intervention draws on patient, community and health systems learning collaboratives to understand system, clinician, lay health worker and patient priorities and how best to implement the intervention. Its systems strengthening and patient-facing elements are accompanied by a clinical component. This comprises a clinical decision support tool and health educational materials for use by PHC clinicians during consultations with people with MLTCs. ENHANCE intervention components appear in box 1.

Box 1 **Components of the ENHANCE intervention**

The ENHANCE intervention aimed to support integrated clinical care for multiple long-term conditions (MLTCs) while encouraging task-sharing among the primary care team to assist with screening, monitoring and health education activities to streamline the offering for the patient and lessen clinician load.^47^ This was informed by patient and community pathways, which mapped long-term condition care at facilities and in communities. The intervention comprised the following components:

- ENHANCE guide: clinical decision support tool for use in a primary care MLTC consultation.
- A long-term condition screening and monitoring poster for screening stations that structured activities that could occur before the consultation.
- Health education posters for health facility waiting rooms. These focused on key health promotion messages, patients’ system literacy and their role as participants in the health system.
- Health education talks accompanied the posters and were delivered by health promotion officers and lay health workers.
- Lay health worker home visits to patients with MLTCs.
- A patient-held ‘Personal Health Diary’ to encourage self-management, treatment adherence and lifestyle behaviour change and to serve as a health promotion tool for lay health worker conversations with patients and their families during home visits and at medication pick-up points.
- A medication list to support lay-health worker treatment literacy efforts.

This practice paper forms part of a collection describing PACK’s role as part of health system strengthening reforms in LMICs. It describes the reconfiguration of the PACK guide to become the ENHANCE guide, a clinical decision support tool for MLTC primary care. The purpose was to consolidate the clinical decision-making required for each long-term condition and encourage a person-centred approach to MLTC consultations. The paper is relevant for clinicians and policy-makers wanting to streamline offerings for people with MLTCs, and those crafting clinical guidance to structure that approach.

### Clinical decision support for MLTC care: the ENHANCE guide

The ENHANCE guide is a 40-page document covering the integrated routine care of 11 long-term conditions—hypertension, HIV, diabetes, depression/anxiety, cardiovascular (CVD) risk, ischaemic heart disease, stroke, epilepsy, asthma, COPD and chronic arthritis.

Six sections structure the consultation flow and profile key elements of an MLTC check-up: identifying and addressing patient priorities, checking long-term condition control and for new comorbidities, reviewing medication, promoting self-management and arranging follow-up care.

The guide contains items that prompt identification of long-term condition deterioration, medication side effects and new comorbidities on history, examination and investigations—22 history items, 6 examination items and 14 investigation items. A list of 48 medications aligns with what is available in public-sector PHC. Guidance is given on prescribing rationally, adherence difficulties, behaviour change, effective communication and local resources for clinician and patient. The final section covers referral prompts and linkage to care with the PHC team and community resources. A video developed for the ENHANCE online training package for PHC clinicians outlines the guide features (video 1).

To facilitate usability at point-of-care for users, the guide’s written style and format draw on decision-making tool features of the PACK guide^16^ (table 1).

**Table 1:** Clinical decision-making tool features of the ENHANCE guide

Feature	Rationale for feature	Example
Person-centredness		
Agenda setting with the patient	This encourages the patient to participate in the consultation and makes them feel heard. In the patient with MLTCs (and likely several issues) this is also a mechanism to encourage the patient and provider to focus on priority items in a brief consultation.	‘You are here for your check-up today. Is there a specific issue we need to address at this visit?’
Addressing patient priority as a first step in the consultation	This aims to help the patient feel that the clinician is taking their concerns seriously.	The flow of the guide supports the clinician to address the patient priority first. ‘Address the issue that the patient would like to focus on today.’
Illustrations/photographs	In the agenda setting exercise, illustrations are included to facilitate a discussion with the patient that encourages them to identify the key issue they want to address. In addition, this will be useful in consultations with patients of limited literacy.	‘Feeling unwell?’ is accompanied by a picture of a woman coughing; ‘Too stressed to cope?’ by a man looking stressed with his head in his hands.
Involving the patient in decision-making about health risk screening	This allows the inclusion of the patient’s values and preferences around health risk assessment and care.	‘Decide with the patient which health screens to focus on.’
Prompt to promote use of self-management tool, the ‘Personal Health Diary’	This is a tangible way to support self-management in the MLTC consultation.	‘Use the Personal Health Diary to help the patient take charge of their health.’ The clinician must complete with the patient relevant items on the tool like medications and test results.
Communication	This is a key feature of person-centred care.	A page in the ENHANCE guide, ‘Communicate effectively’, provides practical communication tips.
Standardised format		
Checklists	The list of routine care items to assess during a multiple long-term condition consultation is long. Arranging them in checklist format allows for a quick glance through what is required and facilitates prioritisation of activities.	The ‘Decide what routine care items to address at this visit’ table provides a checklist of items that the clinician and patient must consider during each consultation.
Tables	Arranging clinical content in tables allows for a standardised, easy-to-navigate layout for the items it contains. Users of the PACK guide are familiar with clinical content arranged in table format.	The long-term condition medications are arranged in an eight-page table. The medications are arranged in alphabetical order and have columns that provide the indication, dose, contraindications and cautions (including interactions), when to consider stopping it and likely side effects.
Colour coding of each medication	To delineate the prescribing scope of practice among the various users of the guide	Simvastatin (highlighted blue) may be prescribed by a doctor or a clinical nurse practitioner who is an authorised prescriber.
Shading of condition-specific content	For ease of navigating treatment and chronic condition screening tables	Table rows of HIV-relevant screening and treatment recommendations are shaded orange (similar to the PACK guide).
Writing style		
Concise text	To ensure clarity of clinical recommendations.	‘If CVD risk>20%, give simvastatin.’ (From the Treatment section of the ENHANCE guide.)
Plain language, avoiding jargon	Ensures clarity of recommendation, regardless of cadre of health worker.	‘If BP is <140/90, continue treatment.’ rather than ‘If normotensive, continue treatment.’
Uses active voice to address the user	Makes it clear that the user is responsible for the clinical activity recommended. Also, it is more concise and easier to understand.	‘Assess patient’s contraceptive needs.’ rather than ‘Patient’s contraceptive needs should be assessed.’
Focus on the individual patient	Addressing the clinical scenario of a single patient supports the user to focus on the patient in each consultation rather than considering all patients.	‘If patient smokes, encourage to stop.’ rather than ‘Encourage all smokers to stop smoking.’
Hard copy features		
Ring bound	Aids page turning and place holding.	-
Printed on durable paper	Allows for regular use for the duration of the publication period.	-
Branding		
Look and feel	The look and feel of the guide are similar to that of PACK and this supports its adoption by health workers who trust the PACK guide as a reliable source of up-to-date policy-aligned clinical content.	The cover, layout and font of the ENHANCE guide echo that of the local PACK guide.
Logos	Logos indicate the endorsement by relevant authorities of the ENHANCE guide.	Local government Department of Health, PACK and ENHANCE study logos appear on the cover.

ENHANCE, EvideNce-led co-created HeAlth systems interventioNs for MLTC-M CarE; MLTCs, multiple long-term conditions; PACK, Practical Approach to Care Kit.

### Developing the ENHANCE guide

#### ENHANCE guide development methodology

Three KTU clinical content editors with experience in clinical decision support curation, evidence and policy synthesis and clinical primary care led the ENHANCE guide development. They drew on the expertise of clinicians and managers leading long-term care health system initiatives as well as researchers in the ENHANCE research team. All are coauthors of this paper.

As we were unable to identify an accepted development methodology for clinical decision-making point-of-care tools for MLTC care, the ENHANCE guide development approach followed that used for the PACK guide.^16^ The rationale for deriving decision-making guidance from clinical practice guidelines and policy is to facilitate their implementation. Thus, we considered best practice approaches in the Guideline Implementability for Decision Excellence Model,^28^ which outlines the characteristics of the developers, the content creation process and the communication of that content. As effective communication of clinical content is key to implementability,^29^ we paid attention to the tool’s user-friendliness, focusing on language, design elements and formatting. We also drew on a theoretical framework that identifies elements of people-centred care.^30^

We adopted a coproduction approach to ensure research quality and impact on policy and practice.^31^ We involved stakeholders in all activities of the ENHANCE study and guided development. Learning collaboratives were established in both provinces to cocreate and test a collaborative care model for MLTC care. We convened an advisory board, clinical working group, end-user testing group and lived experience group to ensure that the ENHANCE guide reflected service and clinical priorities for person-centred MLTC care. Table 2 outlines these groups and the contributions made to ENHANCE guide development. We separated people with lived experience of MLTCs from providers to create a forum where patients did not feel intimidated to speak in front of health professionals.^32^ All participants consented to their involvement and advisory board members completed conflict of interest statements.

**Table 2:** Stakeholder groups

Group	Composition	Purpose	Contribution	Number of meetings
Learning collaborative	Health system managers Policy-makers Lead primary care clinicians Pharmacist from the department of health chronic medication dispensing unit Community-based services coordinators 20 participants in Western Cape, 11 in KwaZulu-Natal	Inform the scope of the ENHANCE package. Inform and approve the approach of the ENHANCE guide. Identify and leverage existing resources, processes and strategies.	Contributed to the focus of key messages for the ENHANCE Guide and related materials. Approved the inclusion of a person-centred priority setting upfront in the multiple long-term condition consultation.	Five in the Western Cape; eight in KwaZulu-Natal
Advisory board	Lead clinical editor (chair) (1) Mental health expert (2) Non-communicable diseases expert (1) Communicable diseases expert (1) Provincial Department of Health representatives (3) Health economist (1) Clinical guideline methodology specialist (1) ENHANCE study principal investigator (1) 11 members	To inform the clinical scope of the ENHANCE guide. To contribute to clinical priority-setting decisions. To provide strategic advice to support the KTU team resolve queries pertaining to the content of the package To suggest relevant stakeholders to contribute to the development, review and testing processes. To ensure that the design of the package supports the principles of equitable, people-centred and community oriented primary care.	Approved the choice of the long-term conditions to be featured in the ENHANCE Guide. Approved the approach to integrate the content of 11 long-term conditions rather than focus on specific combinations of multimorbidity. Contributed to and approved elements of a person-centred care framework. Provided relevant policies and resources to inform the package. Suggested managerial, clinician and community-based stakeholder to contribute to the development and testing processes.	Four quarterly meetings
Clinical working group	Primary care health facility managers (4) Family physicians (2) Primary care doctors (5) Clinical nurse practitioners who work as independent clinicians (4) Professional nurses who support delivery of chronic condition care (4) 19 participants	To provide input into the approach and the clinical content of the ENHANCE Guide to ensure that it is user-friendly and speaks to the need, resources and policy requirements of a primary care clinical setting. To inform the rationalisation and location of long-term condition screening and monitoring activities in order to streamline the patient visit. To interrogate the clinical content using case scenarios. To engage in discussions where needed to resolve clinical queries.	Reviewed the clinical content of the ENHANCE guide for accuracy, user friendliness and alignment to need. integrate disease management priorities with pragmatic approaches to primary care. Suggested the addition of epilepsy and chronic kidney disease into the ENHANCE guide.	Three meetings
End-user testing group	Primary care doctors (3) Clinical nurse practitioners who work as independent clinicians (4) Professional nurses who support delivery of chronic condition care (4) 11 participants. All were enthusiastic users of the PACK guide.	Test the approach of the ENHANCE guide in an MLTC consultation or with real patient scenarios. Identify inaccuracies or gaps in the clinical content. Provide feedback on usability of the ENHANCE guide.	Difficulty navigating large tables prompted the introduction of colour-coding of rows by condition for ease of use. Confirmed that the approach was a feasible one, however, expressed concern about the time required to follow the guide thoroughly.	One meeting and feedback via email
Lived experience group	People who are living with combinations of the following long-term conditions: hypertension, diabetes, HIV, arthritis, depression, COPD, asthma 16 participants	Ensure that people with MLTCs contribute to the development of the ENHANCE guide and other materials in the package. Inform the development of key health education messages for integration into clinical content and other materials. Contribute to developing a person-centred approach to the multiple long-term condition consultation.	Emphasised need for condition and treatment literacy in the intervention. Informed and endorsed the usability of the person-centred priority-setting exercise upfront in the consultation. Suggested a focus on chronic pain care in the multiple long-term condition consultation. Flagged that people rarely read text-heavy health education materials.	Three meetings

COPD, chronic obstructive pulmonary disease; ENHANCE, EvideNce-led co-created HeAlth systems interventioNs for MLTC-M CarE; KTU, knowledge translation unit; MLTC, multiple long-term condition; PACK, Practical Approach to Care Kit.

#### ENHANCE guide development

**Figure 1:**
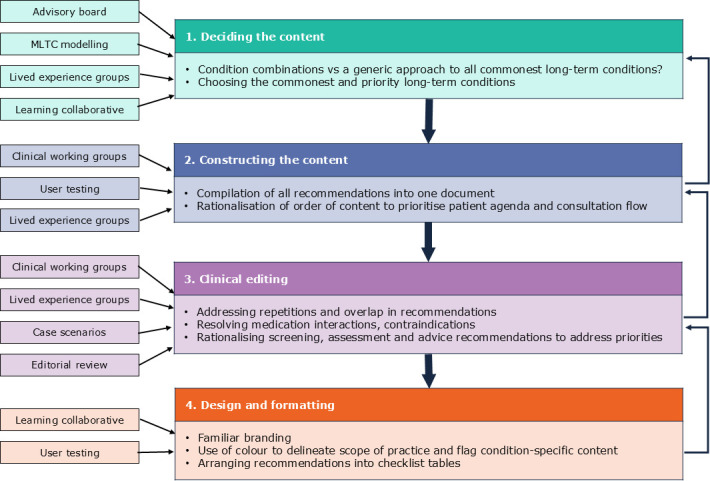
Step-by-step approach to content development including key elements of each step and factors that influenced the approach. MLTC, multiple long-term condition.

The work to develop the ENHANCE guide occurred between September 2021 and May 2023. The process comprised four steps that, while largely sequential, were also iterative as stakeholder input, concomitant research outputs and internal review led to adjustments in each step. Summarised in figure 1, they were (1) deciding the content, (2) constructing the content, (3) clinical editing and (4) design and formatting.

##### Deciding the content

We used the long-term condition content of the PACK Adult Western Cape guide (2021 edition)^33^ as our starting point. Initially, we combined recommendations for the multimorbidity combination of the most common communicable condition (HIV), non-communicable disease (hypertension) and mental illness (depression) while maintaining the PACK guide long-term condition structure. We planned to replicate this approach for the four most common MLTC combinations, but two issues altered this decision. First was a concern that busy clinicians seeing patients with a range of MLTC combinations might forget to consult a guide that only applies to people with specific combinations. Second, the ENHANCE study epidemiological modelling of MLTCs in South Africa showed that hypertension (estimated prevalence: 34%) drives the most common MLTC combinations with arthritis, diabetes and HIV.^3^ Thus, it was more logical to consolidate the content of the most common long-term conditions, knowing that hypertension care (or screening) would underpin almost every long-term condition consultation and ensure that clinicians would use the guide with all patients with MLTCs.

Aiming to develop a guide that could be expanded to become comprehensive, we chose ten common long-term conditions to provide a range of conditions to test the approach. The selection of these conditions was informed by analysis of published and unpublished South African prevalence data.^3^ Table 3 contains the list of 24 PACK guide long-term conditions, and those included in the ENHANCE guide, along with the local prevalence of each. We excluded conditions that require routine care for limited periods, like tuberculosis (TB), pregnancy and palliative care, and less common conditions like peripheral vascular disease, heart failure, fibromyalgia and gout. While the ENHANCE guide included prompts to screen for substance misuse and family planning needs, we referred users to the PACK guide for more detail. In response to Clinical Working Group input, we added epilepsy, which, although uncommon, requires particular guidance for anticonvulsant drug interactions. While chronic kidney disease does not appear as a stand-alone long-term condition in the PACK guide (it is largely specialist managed), PHC-relevant content for screening, identification, referral and pharmacological considerations for that condition was included. The final choice of 11 long-term conditions was endorsed by all stakeholder groups.

**Table 3:** Chronic conditions contained in the PACK and ENHANCE guides along with national prevalence estimates of long-term conditions included in the ENHANCE guide, derived from ENHANCE study modelling^3^

Long-term condition	PACK guide	ENHANCE guide	National prevalence estimates (15 years+)
Tuberculosis	√		0.99% (0.95%–1.04%)
HIV	√	√	18.2% (17.5%–18.6%)
Asthma	√	√	3.5% (3.1%–4.0%)
Chronic obstructive pulmonary disease	√	√	1.8% (1.5%–2.2%)
Cardiovascular risk	√	√	
Diabetes	√	√	10.2% (8.9%–11.7%)
Hypertension	√	√	33.9% (31.8%–36.1%)
Heart failure	√		
Stroke	√	√	2.6% (2.0%–3.5%)
Peripheral vascular disease	√		
Ischaemic heart disease	√	√	5.6% (4.5%–6.9%)
Depression/anxiety	√	√	4.9% (3.9%–5.9%)
Schizophrenia	√		
Dementia	√		
Alcohol/drug use	√		
Tobacco smoking	√		
Chronic arthritis	√	√	11.3% (8.9%–13.7%)
Gout	√		
Fibromyalgia	√		
Epilepsy	√	√	
Contraception	√		
Pregnancy	√		
Menopause	√		
Palliative care	√		

ENHANCE, EvideNce-led co-created HeAlth systems interventioNs for MLTC-M CarE; PACK, Practical Approach to Care Kit.

##### Constructing the content

This step involved compiling PACK guide recommendations for all 11 conditions into one document.

As we aimed for a more person-centred approach, this also required reconfiguration of the content to encourage patient participation in clinical decision-making to address their priorities, rationalise the plethora of check-up activities and support self-management.^34^

A review of PACK history-taking items revealed five featured in each routine check-up no matter the long-term condition. These were symptoms, adherence issues, social problems, mental health concerns and substance use issues. This created a simple approach to agenda-setting with the patient. We included images to depict each one so the page could be used as a discussion tool, with the clinician prompting the patient to pick the issue they needed to address at that visit. The PACK guide uses images to support behaviour change discussions around CVD risk and mental health. A waiting room poster echoed these five images, asking ‘What is the most important thing you would like to talk about today?’—preparing the patient for agenda-setting and permitting the clinician to focus on their agenda. This element was welcomed by stakeholders.

This approach to history-taking meant that routine, active screening for symptoms that would flag a common comorbidity (eg, TB in HIV) or deterioration in a known long-term condition (eg, transient ischaemic attack in hypertension) still needed to be included because these might not be volunteered by the patient. This formed the next section of the guide.

We consolidated the examination and investigation recommendations for all 11 conditions into tables that allowed the user to select the items relevant to the patient’s MLTC combination. We placed general health screening recommended for every adult regardless of long-term condition (eg, cervical screening) as a ‘mop-up’ activity at the end of the consultation, once all long-term condition routine care was completed. This was informed by a process evaluation of PACK guide use in paediatric PHC that demonstrated the screening load often distracted the clinician from the patient’s presenting issue.^21^ However, the clinical working group and end-user input suggested adding this component into the history-taking, examination and investigations section would suit the consultation flow. We structured the treatment section into an overview of the treatment approach (pharmacological and non-pharmacological) for each condition and a table of medications arranged alphabetically with information about when to prescribe, dose, contraindications, considerations around stopping and common side effects.

##### Clinical editing

Once the content was constructed, we ensured its clinical accuracy and attempted to limit the number of recommendations. We removed duplicates, addressed medication interactions and contraindications, resolved the overlap of symptomatology between conditions and medication side effects and ensured consistency in test result thresholds. For example, in the PACK guide, referral thresholds for renal dysfunction differed between HIV (estimated glomerular filtration rate (eGFR) of 60 mL/min/1.73 m^2^) and hypertension (eGFR of 50 mL/min/1.73 m^2^).

Two additional KTU clinical content editors (previously uninvolved in the ENHANCE work) then reviewed for clinical accuracy and to ensure that recommendations had not been omitted or distorted in the transition from PACK. This identified omission of influenza and COVID-19 vaccine recommendations (highly relevant in MLTC care) and flagged discrepancy in amlodipine dosing recommendations for patients with both hypertension (5 mg at night) and ischaemic heart disease (5 mg in the morning). The construction of training case scenarios exposed further gaps and inconsistencies, prompting another round of clinical editing.

##### Design and formatting

Design and formatting ensured that the guide was user-friendly and familiar to users who already knew the PACK guide, incorporating trusted features of the brand, such as font, colour and tables (see table 1).

### Challenges and lessons

A lack of recognised methodology for developing point-of-care tools for MLTC in an LMIC context meant that we adopted an iterative approach that drew on several frameworks for guidance development and our experience of developing the PACK guide. Having the policy-aligned and evidence-aligned PACK content curated separately in a standard structure for 24 conditions enabled its reconfiguration into a consolidated approach for MLTC care. The ENHANCE guide development process contributes to the literature on methodologies for clinical decision support development.

The key challenge was sifting through the large volume of clinical content for 11 chronic conditions to produce guidance that addresses the complexity of multimorbidity safely but remains usable at point of care. Attempts to simplify recommendations revealed tensions around prioritising one condition over another, curative over preventive treatment or pharmacological therapies over advice-giving. For example, given limited evidence for clinician advice-giving around physical activity improving cardiovascular or mental health,^35^ downplaying its inclusion may be justified. However, our attempts to reduce the volume of content of the ENHANCE guide to make it more user-friendly largely failed. This was because clinicians (generalist and specialist) and policy-makers were reluctant to make the tough decisions to omit recommendations, such as depression screening in all patients, which while a common comorbidity,^36^ can be time-consuming^37^ and is often neglected.

The likely disparity between patient and clinician priorities for long-term care^38^ highlights the challenge of navigating between the volume of clinical content and making the consultation person centred. This is a particular issue in low-resource settings which (outside of HIV and TB care) largely lack health management information system support to deliver reminders about routine screening and monitoring, thus placing this aspect of MLTC care at the centre of the PHC consult.

Attempting to adopt a coproduction approach to the guide’s development strengthened the process and the end product, by ensuring key decision-maker input, alignment with policy and other health systems initiatives and drawing on knowledge and experience of both patients and providers.^39^ However, it presented its own challenges. Although we had established relationships with the departments of health and other stakeholders, time pressures and competing priorities meant variable engagement. We mitigated this by alternating face-to-face meetings (which nurtured buy-in into the project) with online and email engagements. The same constraints meant that we struggled to convene cross-provincial engagements, which might have strengthened generalisability of the intervention and allowed for shared learning.

### Recommendations and next steps

To our knowledge, there is no other clinical guidance available that provides detailed clinical decision-making support for an MLTC consultation in an LMIC primary care setting. The ENHANCE guide combines features that may be key elements to support clinicians and patients: its comprehensive content is integrated for 11 conditions that contribute to common MLTC clusters, it provides a person-centred structure that encourages priority-setting with the patient, it is policy-aligned and tailored to be integrated alongside other health system initiatives.

Further work is needed to limit the volume of recommendations in the ENHANCE guide to allow clinicians and patients to focus on patient priorities and the key activities that mitigate the impact of the person’s MLTCs.^40 41^ Currently, much literature and guidance on priority setting in MLTCs is framed in a high-income setting where people with MLTCs are older, often frail and dealing with an end-of-life scenario, which influences their choices and priorities around care.^42^ The context differs in LMICs as MLTCs more commonly occur in younger populations,^43^ leaving a gap in methodologies for priority setting in this context.

Alongside the PACK guide, there is an evidence base and localisation model that provides a template and mechanism for adaptation to other low-resource settings.^42 44^ It could provide a feasible approach to support the uptake of the ENHANCE guide elsewhere.

Advancements in technology offer several solutions to making consolidated up-to-date clinical content easily available in an MLTC consultation.^45^ End-user groups expressed interest in an ENHANCE guide app, which might support usability and diminish the volume of content to navigate per patient. A prototype is being piloted alongside the ENHANCE study. The KTU is also exploring the potential of artificial intelligence to streamline updating processes. We have yet to consider how best to integrate MLTC guidance into electronic health record systems to support timely screening and monitoring.

The ENHANCE study is assessing the effectiveness of the ENHANCE intervention on detection of comorbidity, improvement in detection, treatment and control of long-term conditions, health-related quality of life and functioning, healthcare utilisation and adherence, and the costs of healthcare in people with MLTCs attending PHC services. A parallel process evaluation will explore the ENHANCE guide’s reach, effectiveness, adoption, implementation and maintenance (REAIM).^46^ If proven effective, additional long-term conditions could be added to the ENHANCE guide to provide an expanded offering to support MLTC care.

## Data availability statement

All data relevant to the study are included in the article or uploaded as online supplemental information.
